# Dirofilaria repens infection of the eye

**DOI:** 10.1186/s12348-022-00290-6

**Published:** 2022-03-24

**Authors:** Karl Engelsberg, Jonas Bläckberg

**Affiliations:** 1 Department of Ophthalmology, Clinical Sciences, Lund University and Skåne University Hospital, Ögonklinik B, Klinikgatan 18, SE-221 85 Lund, Sweden; 2 Department of Infectious Diseases, Clinical Sciences, Lund University and Skåne University Hospital, Lund, Sweden

## Case report

A 43-year-old woman was referred to the Eye clinic at Skåne University Hospital complaining of redness and a foreign body sensation in the left eye for 2 to 3 days. Vision was unaffected, and she was otherwise completely well. In particular, there was no itching or swelling elsewhere.

Five months previously, she had visited Pondicherry in India, where she stayed for 3 months. There was no other relevant travel history. Uncorrected visual acuity was 6/6 in both eyes.

**Fig. 1:**
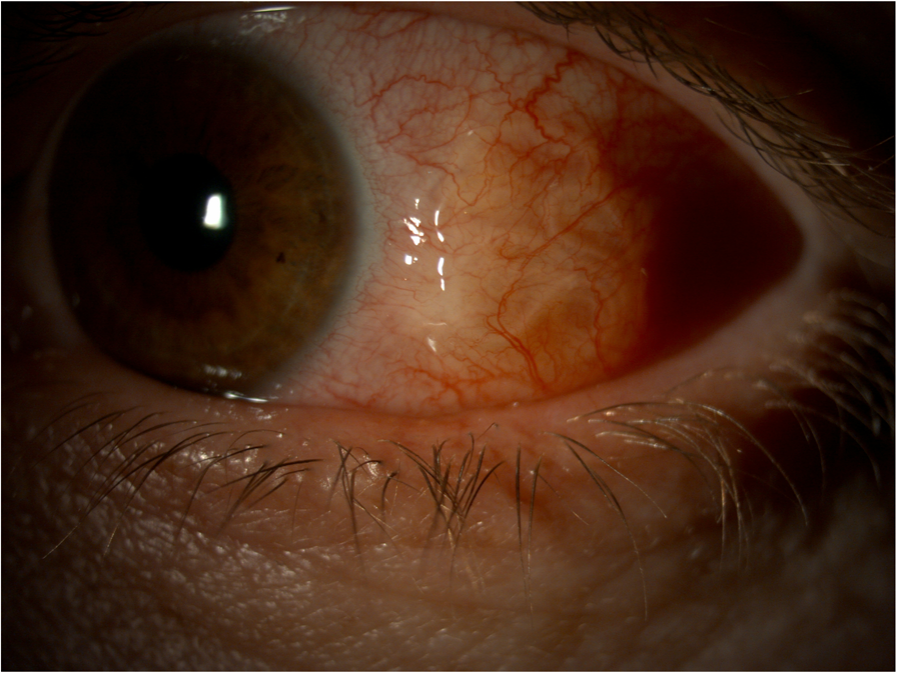
The worm is seen in the temporal part of the bulb under the conjunctiva

On biomicroscopy, we found a motile subconjunctival roundworm in the temporal aspect of her left eye (Fig. 1 and film 1). The overlying conjunctiva was injected, but the remaining eye structures were completely normal, with no other inflammation inside the eye.

She consented to have the worm removed in the surgical theatre and was operated shortly afterwards (film 2). The conjunctiva was incised, and the worm was easily removed in its entirety with forceps. The conjunctiva was closed with 8/0 resorbable sutures (Vicryl® Rapide, Ethicon). Chloramphenicol ointment (Chloromycetin® 1%, Pfizer) was applied three times daily for one week.

**Fig. 2:**
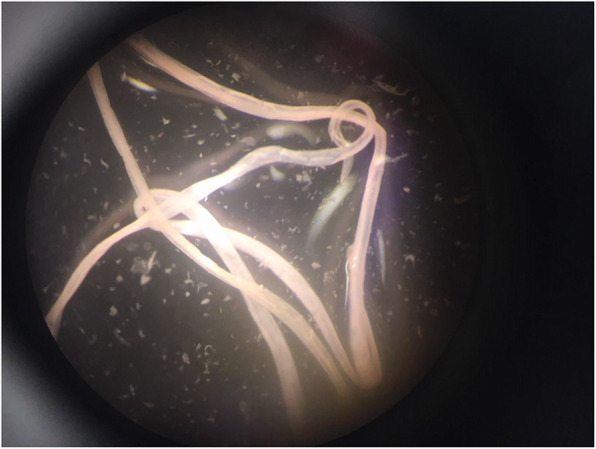
The nematode placed in a vial after removal from the subconjunctival space

The nematode worm, measuring 10 cm in length (Fig. 2), was placed in a vial containing 70% Ethanol and sent for analysis including PCR. Blood tests were requested for filaria serology and eosinophilia.

The microscopic appearance and PCR analysis confirmed it to be an adult female Dirofilaria repens nematode. An immunological assay (ELISA) for filarial antibodies was performed, and this was strongly positive. Her eosinophil count was normal.

One week later, the conjunctiva had healed well and the rest of the eye was unremarkable. Our patient remains completely well.

*D. repens* is a filarial nematode which primarily infects domestic and wild canids, and occasionally felids. Culicine and anopheline mosquitoes are the usual vectors [1]. Third-stage larvae are introduced onto the skin during a blood meal from an infected mosquito, where they penetrate the wound to reach the subcutaneous tissue [2].

Humans are aberrant hosts in which *D. repens* larvae can sometimes reach the adult stage. Typically, infections in humans manifest as a single subcutaneous nodule in exposed sites such as the face. The nodules containing a worm, which may migrate through the tissue causing localised swelling and tenderness [3]. Although they are not thought to reach sexual maturity in the majority of cases, there are sporadic reports of microfilariae found in the peripheral blood [4]. Moreover, human pulmonary dirofilariasis has been reported with this species [5], implying migration of larvae through blood vessels to distal sites. The adults do not survive long, but may cause embolisation. Intra- and periocular tissues can be involved [6–8], as well as male genitalia and the central nervous system [3, 9]. Other Dirofilaria species capable of invading ocular tissues include *D. immitis* and *D. tenuis*.

It was not possible to ascertain the precise pathway from inoculation to adulthood in this case, but we speculate that a mosquito bite close to the site was responsible.

*D. repens* is endemic to the Old World, with the highest incidence of human cases thought to be in the Mediterranean [10]. Its geographic range has expanded in recent years as far north as Finland, probably due to increased movement of infected animals and changes of vector endemicity [11].

This rare case highlights the possibly increasing incidence of human dirofilariasis. We cannot know with certainty where the infection with the nematode was acquired, but initially symptomless infection in a remote tourist destination may be a possibility. A latent period of approximately five months between inoculation and maturation of the worm is plausible, highlighting a risk long after the patient left the endemic region.

We found one previous recording of the removal of the *D. repens* from the eye, however, that recording was rather blurry [12]. Our film emphasizes the relative ease by which it can be extracted in its entire length from the subconjunctival space. In conclusion, we report the first incidence of a live subconjunctival *D. repens* worm in the Nordic countries.

## Supplementary Information

**Additional file 1.**

**Additional file 2.**

## Data Availability

The datasets used during the current study are available from the corresponding author on reasonable request.

## References

[1] Otranto D, Dantas-Torres F, Brianti E, Traversa D, Peric D, Genchi C, Capelli G (2013). Vector-borne helminths of dogs and humans in Europe. Parasit Vectors.

[2] Haim A, Kitchen M, Auer H, Rettenbacher T, Schmuth M (2020). A case of human Dirofilaria repens infection, causing an asymptomatic subcutaneous nodule. Parasitol Res.

[3] Pampiglione S, Rivasi F, Angeli G, Boldirini R, Incensati RM, Pasormerlo M (2001). Dirofilariasis due to Dirofilaria in Italy, an emergent zoonosis: report of 60 new cases. Histopathology.

[4] Potters I, Vanfraechem G, Bottieau E (2018). Dirofilaria repens nematode infection with microfilaremia in traveller returning to Belgium from Senegal. Emerg Infect Dis.

[5] Rena O, Leutner M, Casadio C (2002). Human pulmonary dirofilariasis: uncommon cause of pulmonary coin-lesion. Eur J Cardio Thorac Surg.

[6] Frenzen FS, Loewe I, Müller G, Schoenlebe J, Tappe D, Teichmann D (2021). Dirofilaria repens infection of the eye with concomittant microfilaremia in traveller. J Travel Med.

[7] Mittal M, Sathish KR, Bhatia PG, Chidamber BS (2008). Ocular dirofilariasis in Dubai, UAE. Indian J Ophthalmolol.

[8] Lippera S, Ferroni P, Lippera M (2020). Ocular filariasis via Dirofilaria Repens in a fibrotic, filtering bleb. JAMA.

[9] Poppert S, Hodapp M, Krueger A, Hegasy G, Niesen W-D, Kern W, Tannich E (2009). Dirofilaria repens infection and concomitant meningoencephalitis. Emerg Infect Dis.

[10] Capelli G, Genchi C, Baneth G, Bourdeau P, Brianti E, Cardoso L, Danesi P, Fuehrer HP, Giannelli A, Ionică AM, Maia C, Modrý D, Montarsi F, Krücken J, Papadopoulos E, Petrić D, Pfeffer M, Savić S, Otranto D, Poppert S, Silaghi C (2018). Recent advances on Dirofilaria repens in dogs and humans in Europe. Parasit Vectors.

[11] Pietikäinen R, Nordling S, Jokiranta S, Saari S, Heikkinen P, Gardiner C, Kerttula A-M, Kantanen T, Nikanorova A, Laaksonen S, Lavikainen A, Oksanen A (2017). Dirofilaria repens transmission in southeastern Finland. Parasit Vectors.

[12] Otranto D, Eberhard M (2011). Zoonotic helminths affecting the human eye. Parasit Vectors.

